# Frequency of foot ulcers in people with type 2 diabetes, presenting to specialist diabetes clinic at a Tertiary Care Hospital, Lahore, Pakistan

**DOI:** 10.1186/s12902-018-0282-y

**Published:** 2018-08-06

**Authors:** Bilal Bin Younis, Adeela Shahid, Rozina Arshad, Saima Khurshid, Muhammad Ahmad, Haroon Yousaf

**Affiliations:** 1 Sakina Institute of Diabetes and Endocrine Research, Shalamar Hospital, Lahore, Pakistan; 2 Physiology Department, Shalamar Medical and Dental College, Lahore, Pakistan

**Keywords:** Type 2 diabetes, Foot ulcer, Peripheral neuropathy

## Abstract

**Background:**

Diabetic foot ulceration is a serious limb-threatening complication of diabetes. It is the common cause of hospital admissions and amputations.

The objective of the study was to determine the prevalence of diabetic foot ulcers (DFU) and its association with age, gender, duration of diabetes, peripheral neuropathy (PN), peripheral arterial disease (PAD) and HbA1c.

**Methods:**

A total of 1940 people (≥ 30 years of age) with type 2 diabetes coming to the Sakina Institute of Diabetes and Endocrine Research (specialist diabetes clinic) at Shalamar Hospital, Lahore, Pakistan, were recruited over a period of 1 year from January 2016 to January 2017. The foot ulcers were identified according to the University of Texas classification. PN was assessed by biothesiometer and PAD by ankle-brachial index (< 0.9). Body weight, height, body mass index (BMI), HbA1c and duration of diabetes were recorded.

**Results:**

The prevalence of DFU was 7.02%, of which 4.5% of the ulcers were on the planter and 2.6% on the dorsal surface of the foot; 8.5% of the persons had bilateral foot ulcers and 0.4% subjects had Charcot deformity. There was significant association of foot ulcers with age, duration of diabetes, HbA1c, PN and PAD, whereas no association was observed with gender and BMI. PN and PAD were observed in 26.3 and 6.68% of people with diabetes respectively. Neuropathic ulcers and neuro-ischemic ulcers were identified in 74 and 19% of the study population. Logistic regression analysis revealed significant odds ratio for peripheral neuropathy 23.9 (95% confidence interval (5.41–105.6).

**Conclusions:**

Peripheral neuropathy is the commonest cause of foot ulcers. An optimum control of blood glucose to prevent neuropathy and regular feet examination of every person with diabetes may go a long way in preventing foot ulceration.

## Background

Diabetes mellitus is one of the most prevalent endocrinopathies associated with a number of complications. It is now reaching epidemic proportions in the world, according to the International Diabetes Federation (IDF) 2017 estimates, 425 million people in the world has diabetes and is expected to increase to 629 million in the year 2045. According to the IDF 6.94% of the population of the Pakistan has diabetes and by the year 2045 it is likely to increase to 8.45% [1].

In people with diabetes, lower limb complications are becoming a major public health concern and the incidence is increasing both in developed and developing countries of the world [2, 3]. Foot ulcers are a serious limb-threatening complication of diabetes mellitus [4]. Diabetic foot ulcer (DFU) is defined as the presence of full-thickness lesion distal to the ankle [5]. The global prevalence of DFU was 6.3% and it was more common in people with type 2 diabetes [6] Microvascular and macrovascular complications, peripheral neuropathy, and duration of diabetes mellitus, high levels of HbA1C and trauma are considered as factors contributing to the risk of foot ulcer [7]. In people with diabetes, peripheral neuropathy (PN) results in loss of perception of pain and propioception by the patient, repeated trauma to the foot may not be perceived by the patient and may give rise to symptoms which include formation of callus on weight bearing areas which can lead to development of ulcers [8]. Neuropathic ulcers are the most common type of ulcers resulting from tissue-damage due to mechanical weight applied to a foot with loss of propioception, touch, vibration, and pressure and pain sensation [9].

One of the known macrovascular complications of diabetes mellitus is damage to blood vessels resulting in peripheral arterial disease (PAD). There is high prevalence of PAD in diabetics which can predispose to the formation foot ulcers [10]. Poor control of blood glucose level impairs the cross linking of collagen and functioning of matrix metalloproteinase which adversely affects wound healing [9]. Hyperglycemia also results in poor functioning of polymorph nuclear leukocytes, which predisposes to onychomycosis and toe-web infections, and ultimately results in damage to the skin [11]. Diabetic foot lesions now considered to be the most common cause of hospital admission and lower limb amputation than any other complication of diabetes, screening all patients with diabetes is of immense importance to identify the subjects at risk for developing foot ulcers [12, 13]. There are ethnic differences in the risk of foot ulcer, as South Asians and African-Caribbean have low foot ulcer risk to that of Europeans [14]. In Pakistan 11.77% of the population had type 2 diabetes mellitus [15], as the number of people with diabetes mellitus is increasing, it is likely to increase the burden of diabetic foot disease in Pakistan. The objective of the current study was to determine the prevalence of foot ulcer in diabetics presenting to specialist diabetes clinic at a tertiary care hospital and its association with age, gender, BMI, duration of diabetes, PN, PAD and control of blood sugar level.

## Methods

### Data collection

A total of 1940 people with diabetes (37% males and 63% females) presenting to a specialist diabetic clinic, Sakina Institute of Diabetes & Endocrine Research (SiDER) center at Shalamar Institute of Health Sciences Lahore Pakistan, were included in the study. It was a cross sectional study, completed in a duration of 1 year. Ethical approval of the study was taken from Institutional Review Board (IRB) of Shalamar Medical and Dental College Lahore Pakistan (IRB no: SMDC/IRB/01–08/634). Written informed consent was obtained from all the participants of the study. Diabetic condition was confirmed by review of medical record and previous laboratory tests. Complete computerized demographic information (name, age, sex, address, education, duration of diabetes etc) was recorded. Subjects with type 1 diabetes, gestational diabetes, cognitive impairment and hypothyroidism were excluded from the study.

### Examination

All subjects underwent a detailed general physical and systemic examination. Dorsalis pedis and posterior tibial arteries were examined through palpation.

### Body height & weight

Body weight and height of each subject was measured using digital scale and a wall-mounted stadiometer respectively. BMI of each subject was calculated and subjects were classified in three groups, normal body weight, over weight and obese according to Asian criteria for BMI [16].

### Ankle-brachial index test

Ankle-brachial index (ABI) test was used to determine the PAD. ABI is the ratio of blood pressure at the ankle to the blood pressure in the arm. Systolic blood pressure is measured at the ankle in dorsalis pedis, and at the arm in the brachial artery, by using hand Doppler with a frequency of 8 mHz, while a person is at rest and ratio is taken, ABI > 0.9 is taken as normal and the subject with ABI < 0.9 is diagnosed to have PAD [17, 18]. When ABI is between 0.4 and 0.9 the subjects is having mild to moderate PAD, subject is considered to have severe PAD when ABI is > 0.4 [18].

### Diabetic foot examination

Complete examination of feet was carried out which consisted of skin, musculoskeletal, neurological and vascular examination. Both dorsal and planter surfaces were examined for presence of any ulcers. Staging and grading of foot ulcer was done according to the university of Texas classification [19]. Diagnosis of Charcot foot was based on presence of swelling, redness, increase in temperature and any deformity of muscles and bones of foot which was also confirmed by X-rays.

University of Texas classification of foot ulcer [19]

**Table:** 

Stages	Description
Stage A	No infection or ischemia
Stage B	Infection present
Stage C	Ischemia present
Stage D	Infection and ischemia present
**Grading**	
Grade 0	Epithelialized wound
Grade 1	Superficial wound
Grade 2	Wound penetrates to tendon or capsule
Grade 3	Wound penetrates to bone or joint

### Assessment for peripheral neuropathy

Feet of all subjects were examined for presence or absence of PN by biothesiometer. To measure the sense of vibration subject was first made familiar with the sensation by applying biothesiometer probe on distal palmar surface of hand. Sense of vibration was checked at five locations i.e. distal planter surface of great toe, proximal planter surface of great toe, lateral surface of great toe, planter surface of digiti minimi and planter surface of calcaneus. A biothesiometer probe was held with a constant and firm pressure perpendicularly at each location and voltage was gradually increased till the subject can feel the vibration sensation, the voltage at which patient first felt the vibration sensation is called vibration perception threshold (VPT). This process was repeated three times and mean of readings was taken, subject with VPT of > 25 V was considered as having PN and is a predictor of foot ulceration [20, 21].

### Analytical determinations

Three ml of venous blood was drawn from the cubital vein. HbA1-C was determined in duplicate using high affinity liquid chromatography with a D-SI Glycomat (Provalis Diagnostics, Deeside, UK).

### Statistical analysis

SPSS (Statistical Package for Social Sciences) version 17.0 [SPSS, Inc. Chicago, IL, USA] was used to enter and analyze data. The Quantitative variables were expressed as mean ± standard deviation (S.D), whereas qualitative data was presented by frequency and percentages. Un paired ‘t’ test was applied to determine the significant difference between the groups. The Logistic regression analysis was done to determine the association of age, gender, duration of diabetes, BMI, HbA1-C, PN and PAD with DFU. A *p*-value of < 0.05 was considered statistically significant.

## Results

### Characteristics of study population

A total of 1940 people with diabetes, both males (37%) and females (63%) were included in the study. The mean age, BMI, HbA1-C and duration of diabetes of the study population are given in Table 1. The mean age of females with diabetes was 49.05 ± 10.08 years whereas for males was 52.76 ± 11.31 years. Mean BMI of all subjects included in the study was 23.26, bilaterally symmetrical PN was present in 26.30% and PAD was present in 6.68% of the type 2 diabetic subjects included in the study. Charcot foot was identified in 0.4% of the study population (Table 1).

**Table 1:** Basic characteristics of study population

Parameters	
Total Sample Population (n)	1940
Age (mean ± SD)	51.24 ± 10.60
Gender	
Male n(%)	718 (37.0%)
Age (mean ± SD)	52.76 ± 11.31
Female n(%)	1222 (63.0%)
Age (mean ± SD)	49.05 ± 10.08
Diabetics < 40 years of age	260 (13.4%)
Duration of Diabetes (mean ± SD)	7.29 ± 6.1
BMI (mean ± SD)	23.26 ± 4.59
HbA1c %(mean ± SD)	9.58 ± 2.199
Peripheral Arterial Disease(PAD)	(129) 6.68%
Peripheral Neuropathy (PN)	(509) 26.30%
Foot Ulcers	(136) 7.02%
Charcot foot	(8) 0.4%

### Diabetic foot ulcer

The prevalence of DFU was 7.02% in the study population. Foot ulcers were further divided according to the location, 61.22% of the ulcers were on planter surface as compared to dorsal surface (30.80%), whereas 8.08% of ulcers were both on planter and dorsal surfaces of foot. Neuropathic, neuro-ischemic and ischemic ulcers were observed in 74, 19 and 7% of the people with diabetes (Table 2).

**Table 2:** Location of foot ulcer in people with type 2 diabetes

Parameter	Frequency (%)
Ulcer on Dorsal Surface	30.80%
Ulcer on planter Surface	61.22%
Both Planter and Dorsal Surface	8.08%
Neuropathic Ulcer	74%
Neuro-ischemic Ulcer	19%
Ischemic Ulcer	7%

The mean age (53.73 ± 9.98) of the subjects with DFU was significantly higher as compared to subjects (50.63 ± 10.67) without DFU. HbA1C of subjects with DFU was significantly (*p* < 0.05) higher compared to those with no foot ulcer. There was significant difference (p < 0.05) in the mean duration of diabetes of the subjects with and without foot ulcer, duration of diabetes of the subjects with DFU was significantly more compared to those with no DFU, there was no significant difference (*p* > 0.05) in the BMI of the subjects with and without foot ulcer (Table 3).

**Table 3:** Characteristics of the diabetic subjects with and without Foot Ulcer

Variables (mean ± SD)	Subjects With DFU	Subjects Without DFU	*P* value
Age	53.73 ± 9.98	50.63 ± 10.67	0.024*
BMI	23.75 ± 4.20	23.24 ± 4.61	0.390
HbA1c	10.30 ± 2.09	9.56 ± 2.20	0.001*
Duration of diabetes	9.96 ± 6.35	7.08 ± 6.26	0.001*

*P*-value of < 0.05 was considered statistically significant

### Frequency of foot ulcer with respect to age, BMI, HbA1C and duration of diabetes

When the data of subjects with DFU was further stratified according to age groups, BMI, HbA1C levels and duration of diabetes, 88.7% of the subjects with DFU were more than 45 years of age, no ulcers were observed in age group less than 35 years of age. When subjects were analyzed according to BMI into obese, over weight and normal weight groups, it was observed that 45% of the subjects with foot ulcers were obese and 21% of the subjects were overweight. 60% of the subjects with foot ulcers had HbA1C greater than 10, 35% of the subjects with foot ulcer had HbA1c levels between 7 and 10%. 75.8% of the subjects with foot ulcer had diabetes for more than 6 years and 19% of the subjects had diabetes for less than 6 years (Table 4).

**Table 4:** Frequency of subjects with foot Ulcer with respect to Age, BMI, HbA1C and duration of Diabetes

Age	Foot Ulcers (%)	BMI	Foot Ulcers (%)	HbA1c	Foot Ulcers (%)	Duration of Diabetes	Foot Ulcers (%)
< 35 yrs	0%	Normal	34%	< 7	4.8%	< 1 yrs	5.10%
35–44 yrs	11.3%	Over weight	21%	7.0–10.0	35.5%	1-6 yrs	19.10%
> 45 Yrs	88.7%	Obese	45%	> 10	59.7%	> 6 yrs	75.80%

### Association of diabetic foot ulcers with measured variables

Logistic regression analysis revealed significant association of DFU with age, duration of diabetes, HbA1C, PN and PAD whereas no significant association was observed with gender or BMI. Logistic regression analysis revealed significant odds ratio of 23.9 (95% confidence interval 5.41–105.6) for peripheral neuropathy (Table 5).

**Table 5:** Association of diabetic foot ulcers with measured variables

Variables	P-Value	Odds ratio	Lower	Upper
Age	0.025*	1.027	1.003	1.051
Peripheral neuropathy	0.001*	23.926	5.41	105.6
Peripheral vascular disease	0.001*	0.267	1.43	0.532
HbA1c	0.001*	6.187	4.646	8.239
Duration of diabetes	0.001*	1.063	1.027	1.100

P-value of < 0.05 was considered statistically significant

## Discussion

In the present study 13.4% of the diabetics, presenting to specialist diabetes tertiary clinic were less than 40 years of age. This shows that type 2 diabetes is now occurring at a younger age in Asian population. The frequency of females presented to diabetic clinic was more as compared to males in the present study; however a study carried out in same region has reported the prevalence of 11.20% in males and 9.91% in females with diabetes [15]. Significant number of subjects (89%) developed foot ulcers after 45 years of age and 11% of the subjects developed ulcers of the of foot ulcer at a much younger age, this is further enhanced by the significant association of foot ulcer with age, which is consistent with previous data [22].

Mean age of male and females with type 2 diabetes was 53 and 49 years respectively. The present data signifies that females are at risk of developing diabetes at an earlier age as compared to males. PAD and PN are the complications of diabetes predisposing to foot ulcers, 6.68% of the subjects were found to have PAD, and 26% were identified with PN. The PN was a major factor contributing to the risk for foot ulcers in diabetics; this result is consistent with the previous reports emphasizing the importance of PN in foot [23, 24].

Diabetic foot disease is largely preventable; however it is still a significant cause of hospitalization in people suffering from diabetes mellitus. The prevalence of DFU in the present study was 7.02%, in contrast to this previous studies in which the prevalence was less [6, 14, 25]. According to the present data, 61% of the subjects had ulcers on the planter surface, 31% on the dorsal surface of the foot, 75% of the subjects with DFU had more than 6 years of diabetes in present study; the significant association of foot ulcer with duration of diabetes is in accordance with previous data. In contrast to this it has been reported the risk of foot ulceration is not related to duration of diabetes [26]. The current study reported no association of foot ulcer with gender, which is in accordance with previous studies, [27, 28] in contrast to this few studies have reported males are at more risk of foot disease [29, 30] Data is available suggesting that screening all patients with diabetes is of great importance, to recognize those at risk for developing foot ulceration [31]. Heath education of patients, self examination of feet and optimum control of blood glucose is important for prevention of foot ulcers [32]. In depth knowledge and communication among physicians is vital for preventing the serious limb threatening complications of diabetic foot disease. [33].

As the number of people with diabetes is increasing in Pakistan [1], it is likely to increase the burden of diabetic foot disease in this population. Greater part of the world is severely lacking in foot care specialists and centers, Pakistan is also amongst countries where few foot care services are available. Patient’s education regarding self examination of foot, and identifying the diabetics at increased risk of developing foot ulcers, not only at specialist centers but also training the family physician to carry out routine foot examination will be the most valuable approach to prevent the progression to limb amputation. Proper assessment of the DFU and appropriate management ensure better prognosis.

## Conclusions

Prevalence of foot ulcer in people with type 2 diabetes presenting to specialist diabetes clinic at a tertiary care hospital is 7.02% in the current study. Peripheral neuropathy, peripheral arterial disease, female sex, increasing age, increase in duration of diabetes and high levels of HbA1C increases foot ulcer risk in diabetics. An optimum control of blood glucose, proper feet examination of every person with diabetes and management of associated risk factors may go a long way in preventing foot ulceration. It will reduce hospital admissions and lower limb amputations.

## Abbreviations

ABI: Ankle-brachial index
BMI: Body mass index
DFU: Diabetic foot ulcer
IDF: International Diabetes Federation
PAD: Peripheral arterial disease
PN: Peripheral neuropathy
SPSS: Statistical package for social sciences
VPT: Vibration perception threshold
